# Identification of mine water source based on TPE-LightGBM

**DOI:** 10.1038/s41598-024-62413-4

**Published:** 2024-05-31

**Authors:** Man Wang, Jianguo Zhang, Han Li, Bo Zhang, Zhenwei Yang

**Affiliations:** 1 China Pingmei Shenma Holding Group Co., LTD, Pingdingshan, 467000 Henan China; 2 China Pingmei Shenma Group, State Key Laboratory of Coking Coal Resources Green Exploitation, Pingdingshan, 467000 Henan China; 3 Institute of Resources & Environment, Henan Polytechnic University, Jiaozuo, 454000 Henan China; 4 Collaborative Innovation Center of Coal Work Safety and Clean High Efficiency Utilization, Jiaozuo, 454000 China

**Keywords:** Pingdingshan coalfield, Min water hazard, Tree-structured Parson estimator (TPE), Light gradient boosting machine (LightGBM), Intelligent water source identification model, Hydrogeology, Natural hazards

## Abstract

Mine water inrush is a serious threat to mine safety production. It is very important to identify water inrush source types quickly to prevent and control water damage. In this study, the aqueous chemical components Na^+^ + K^+^, Ca^2+^, Mg^2+^, Cl^−^, SO_4_^2−^ and HCO^3−^ of different aquifers in Pingdingshan coalfield were selected as the characteristic values, and the Surface water, Quaternary pore water, Carboniferous limestone karst water, Permian sandstone water, and Cambrian limestone karst water were used as the labels. An intelligent water source discrimination model is proposed by combining data mining, classification models, and reinforcement learning. As outlier data in the samples may interfere with the model recognition ability, the data distribution range was analyzed using box plots, and 20 groups of abnormal samples were excluded. The processed water chemistry data were divided into 80% learning samples and 20% test samples, and the learning samples were fed into a light gradient boosting machine (LightGBM) for training. The tree-structured parson estimator (TPE) obtains the optimal values of the main parameters of LightGBM in a very short time. Substituting the hyperparameters back into the model yields a 13.9% improvement in the accuracy of the model, proving the effectiveness of the TPE algorithm. To further validate the performance of the model, TPE-LightGBM is compared and analyzed with a Random Search-Multi Layer Perceptron Machine (RS-MLP) and Genetic Algorithm-Extreme Gradient Boosting Tree (GA-SVM). The accuracy of TPE-LightGBM, RS-MLP, and GA-SVM is 0.931, 0.759, 0.724 in that order, and the generalization error RMSE is 0.415, 1.05, and 1.313 in that order. The results show that TPE-LightGBM is more advantageous in water source identification and is more resistant to overfitting. By calculating and comparing the information gain of each variable, the contribution of Ca^2+^ is the highest, so it is necessary to pay attention to the change in Ca^2+^ concentration. TPE-LightGBM’s high accuracy and generalization ability have a good prospect for the identification of sudden water source types.

## Introduction

The North China Coalfield is often engaged in deep coal mining because of the depletion of shallow coal resources. With the increase in the depth of coal mining, the underground hydrogeological conditions of the mines are becoming increasingly complex, which seriously threatens the regular production of coal mines^1^. Sudden water hazards in complex hydrogeological environments are common in mines, and they have seriously harmed coal mine safety output. The risk of mine water inrush accidents to coal resources is quite real; they frequently cause the water bursting in mine completely or experience localized seepage, which lowers production and causes financial losses. As a typical North China coalfield, the Pingdingshan coalfield has faced the threat of mine water surges since its construction. Determining the origin of the sudden water is a crucial first step in preventing and controlling sudden water in the mine. Managing mine flooding usually starts with the source of the sudden water. Therefore, rapid and accurate identification of mine water sources is significant for mine water damage prevention and control^2^. The hydrochemical analysis method is based on the variation of geochemical ion concentrations in aquifers and incorporates mathematical methods to create discriminant functions for different aquifers^3^. The six crucial discriminant ions, Na^+^ + K^+^, Ca^2+^, Mg^2+^, Cl^−^, SO_4_^2−^, and HCO^3−^, are more frequently used in water source identification^4^. Commonly used mathematical methods include distance discrimination^5,6^, Fisher discrimination^7,8^, fuzzy evaluation^9,10^, objective combination of weights^11^, and so on. However, when the amount of data is large, the computation of traditional methods will increase, while the accuracy will be difficult to meet the demand for water source identification.

Machine learning is a branch of artificial intelligence that excels in solving classification prediction and regression problems. It involves research in many areas of statistics, probability theory, approximation networks, neural networks, and optimization theory^12–14^. The water source identification problem can be summarized as a multi-label classification problem in machine learning. Combining machine learning methods with water chemistry ions to construct discriminant models is necessary. Many scholars have achieved fruitful results by introducing machine learning methods into the identification of mine water sources, such as Support Vector Machines, Naive Bayes^15^, Back Propagation Neural Networks^16^, Logistic Regression Analysis^17^, and Random Forest^18,19^. All of the above methods can solve most practical problems. However, when the hydrological conditions in the study area are complex and multiple categories of water samples are combined, Multiple-category categorization is not satisfied by Bayes. Artificial neural networks require numerous samples to learn, and the lack of samples will directly affect the fitting effect of artificial neural networks. Determining the Gamma and penalty coefficients for support vector machines may be challenging. To avoid these problems, scholars choose appropriate methods to construct water source discrimination models from feature processing, model enhancement, and model evaluation. For example, Lin et al. used the improved genetic algorithm (IGA) to overcome the shortcomings of Extreme Learning Machine (ELM) in which the thresholds for weights selected at random^20^. The IGA-ELM water source discriminant model is proposed and achieves good accuracy. Li et al. used the genetic algorithm (GA) for iterative optimization of seven parameters of Extreme Gradient Boosted Regression Tree (XGBoost), and the results showed that GA-XGBoost has higher accuracy and generalization ability^21^. Li et al. combined principal component analysis (PCA), K-dimension tree (KD) and K-nearest neighbor (KNN) algorithms to establish a mine water inrush source identification model, which not only reduces the computational complexity, but also overcomes the influence of overlapping information among indicators on the identification results, thus greatly improving the identification accuracy^22^. Yan et al. adopted the Mayfly Algorithm to optimize the LSTM network, which effectively improved the convergence speed and optimization accuracy, and avoided the algorithm falling into the local optimal situation^10^. However, there are still shortcomings: (1) The model construction is cumbersome and thus increases the computational complexity of the algorithm. (2) The discriminant method does not consider the redundancy of information between water chemistry data, which makes the model training time longer. (3) When small and medium-sized samples are input into the model for learning, the model learning is not sufficient, and the accuracy of the training samples is low, which makes the overfitting phenomenon easy to occur.

In summary, this paper proposes a novel intelligent water source discrimination method. The method uses a tree-structured Parzen estimator (TPE) with improved Bayesian optimization to optimize the critical Light Gradient Booster Machine (LightGMB) parameters and the resulting hyperparametric back generation model. LightGMB uses one-sided gradient sampling (GOSS) to exclude samples with small gradients from the point of view of reducing the number of samples, which does away with the requirement for extensive data processing. TPE-LightGMB achieved a high accuracy of 93. 1% in comparison with RS-MLP and GA-SVM. In addition, TPE takes less time compared to other optimization algorithms. The TPE-LightGMB proposed in the article has high efficiency and applicability, which provides a new idea for mine water source identification.

## The theory of method

### Tree-structured Parzen estimator

TPE (Tree-structured Parzen estimator) is a Bayesian algorithm based on tree structure. It focuses on solving global optimization problems for black-box functions^23^. The TPE algorithm can adaptively regulate the extent of the parameter-seeking search space and find the optimal solution in a small number of iterations. Its optimization function is defined as follows:

1 $$ {\text{x}} = \arg \min f\left( x \right){, } $$

where *x* is the space of solutions, *f(x)* is the objective function, and x* is the optimal solution obtained.

The TPE defines p(x|y) through two density functions:

2 $$ { } p\left( {x{|}y} \right) = \left\{ {\begin{array}{*{20}c} {l\left( x \right), y < y^{*} } \\ {g\left( x \right), y \ge y^{*} } \\ \end{array} } \right.{ },{ } $$

where y* is a set threshold, *l(x)* is constructed from xi in the search space, and the rest of the observations construct *g(x)*. *l(x)* corresponds to a loss *f(x*_*i*_*)* that needs to be less than y*, and *g(x)* vice versa.

The Expected Improvement (EI), which serves as the acquisition function, is transformed in the following ways using the Bayesian formula:

3 $$ {\text{EI}}_{{{\text{y}}^{*} }} \, = \, \int\limits_{ - \infty \, }^{{{\text{y}}^{*} }} { \, \left( {{\text{y}}^{*} \, - {\text{ y}}} \right)} \, \frac{{{\text{p}}\left( {{\text{x}}|{\text{y}}} \right){\text{p}}\left( {\text{y}} \right) \, }}{{{\text{p}}\left( {\text{x}} \right)}}{\text{dy}}{.} $$

First simplify the denominator so that *γ* = p (*y* <  y*):

4 $$ {\text{ p}}\left( {\text{x}} \right) = {\gamma l}\left( {\text{x}} \right) + \left( {1 - {\upgamma }} \right){\text{g}}\left( {\text{x}} \right),{ } $$

where *γ* denotes the constant fraction of TPE, partitioned into *l(x)* and *g(x)*, in the range (0, 1).

Second, the molecule can be made simpler:

5 $$ \mathop \smallint \limits_{ - \infty }^{{y^{*} }} \left( {y^{*} - y} \right)p\left( {x{|}y} \right)p\left( y \right) = \gamma y^{*} l\left( x \right) - l\left( x \right)\mathop \smallint \limits_{ - \infty }^{{y^{*} }} p\left( y \right)dy. $$

After substituting Eqs. (4) and (5) into Eq. (1), it is possible to acquire the simplified acquisition function.

6 $$ \begin{gathered} EI_{{y^{*} }} = \frac{{\gamma y^{{^{*} }} l(x) - l(x)\int_{ - \infty }^{{y^{*} }} {p(y)dy} }}{\gamma l(x) + (1 - \gamma )g(x)} \propto \left( {\gamma + \frac{g(x)}{{l(x)}}(1 - \gamma )} \right)^{-1} \hfill \\ \hfill \\ \end{gathered} $$

To maximize EI, the probability of *g(x)* is low while the probability of l*(x)* is high at point x. During the iteration, the solution x* that maximizes EI is returned.

### LightGBM

LightGBM is improved from the tree model GBDT, which has the advantages of faster training rate, lower memory consumption, higher accuracy, and supports distributed processing of large amounts of data. The objective function of LightGBM can be expressed as^24^:

7 $$ X_{LGBM} = \mathop \sum \limits_{i = 1}^{N} l\left( {y_{i} ,\gamma_{i} } \right) + \mathop \sum \limits_{j = 1}^{t} \omega \left( {f_{i} } \right), $$

where $l\left({y}_{i},{\gamma }_{i}\right)$ denotes the difference between the predicted value and the true value. $\sum_{j=1}^{t}\omega \left({f}_{i}\right)$ denotes regularization, which aims to choose a simple predictive function from preventing model overfitting. $\omega \left({f}_{i}\right)$ denotes the complexity of the tree.

8 $$ \omega (f_{i} ) = \alpha T + \frac{1}{2}\beta \sum\limits_{j = 1}^{t} {w_{j}^{2} } , $$

where *T* denotes the number of leaf nodes of the tree, *w* denotes the leaf node output score of each tree, and $\alpha$ denotes the value that controls the number of leaf nodes. $\beta$ is a variable that is used to avoid the output scores of the leaf nodes being too large.

**Figure 1:**
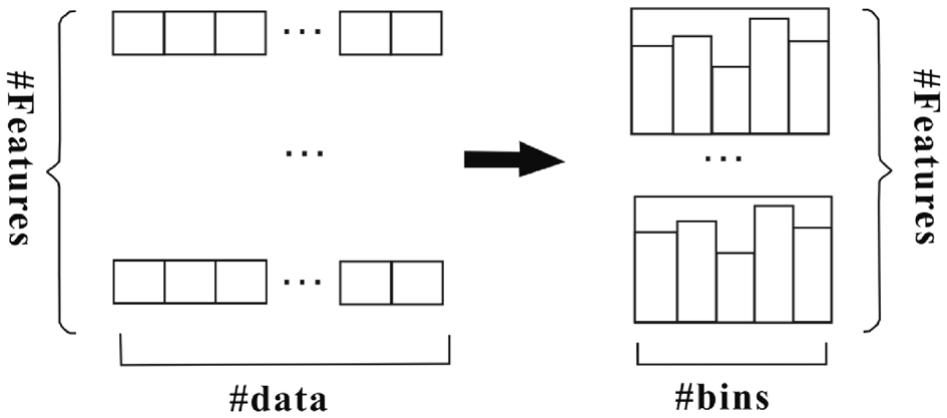
The process of forming a histogram.

LightGBM is constructed similarly to GBDT by iteratively building the optimal decision tree^25^. The biggest difference is that LightGBM uses a histogram algorithm to select features. The principle of the histogram algorithm is that continuous data is first discretized into m integer data. Then the histogram of width m is created. Select the optimal root node in the histogram. When searching for a node once, the histogram accumulates statistics and obtains the optimal segmentation point based on the discrete values of the histogram. The histogram algorithm not only makes the complexity of the model lower but also reduces the memory footprint significantly. The process of forming a histogram by the histogram algorithm is shown in Fig. 1.

In addition, LightGBM uses a one-sided gradient sampling algorithm (GOSS) for sampling. The GOSS algorithm works from the perspective of reducing the number of samples by first eliminating most of the samples with small weights and then calculating the information gain using only the remaining samples. It is an algorithm that balances data reduction with guaranteed accuracy, sufficiently mitigating the time-consuming problem and thus further improving computational efficiency^26^. The classification model constructed in this way is faster and more accurate compared to other gradient models.

## Geological profile

**Figure 2:**
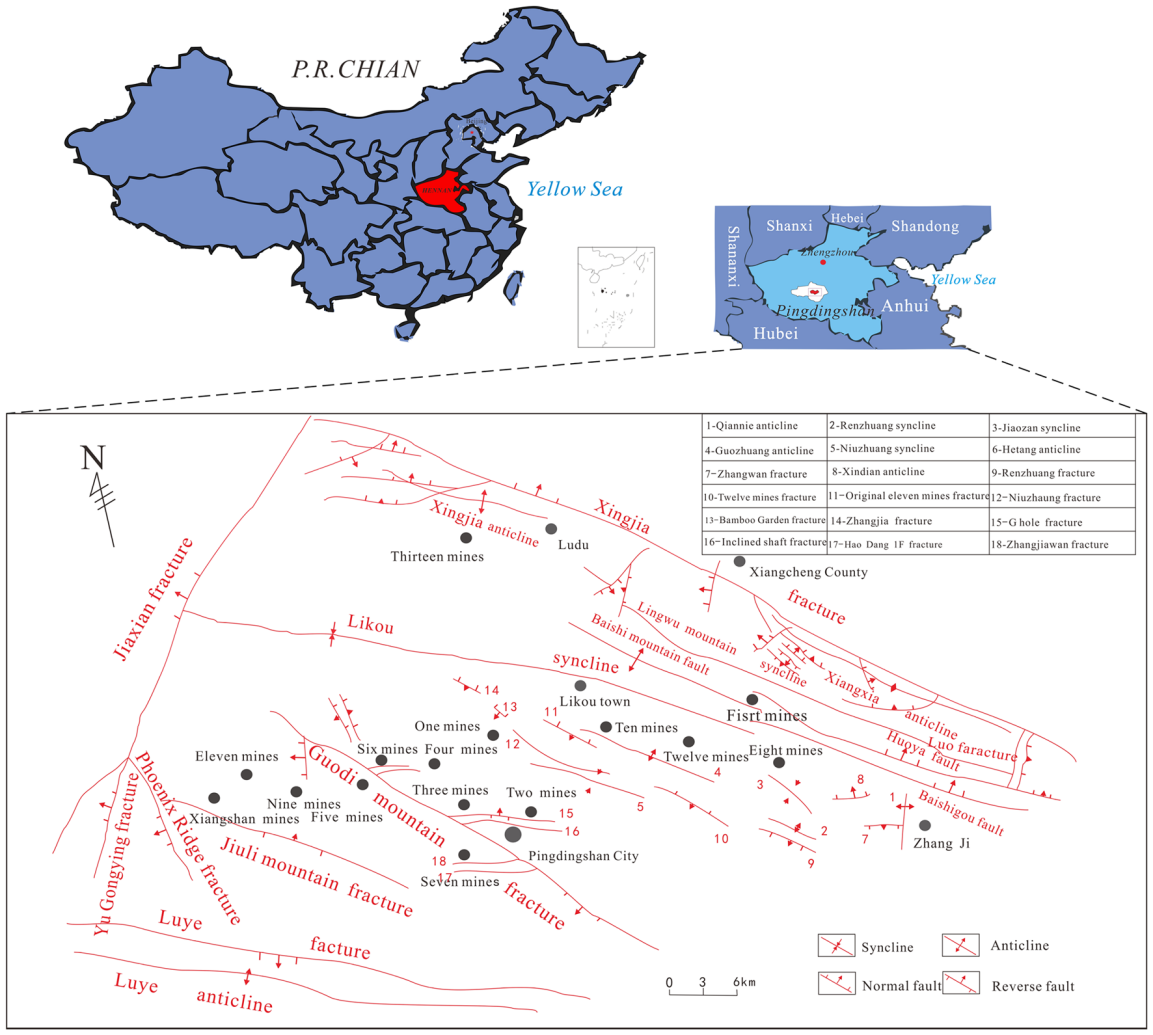
Geological structure map of Pingdingshan coalfield (the figure was drawn by MapGIS 6.7, URL link: https://www.mapgis.com/index.php?a=shows&catid=97&id=29).

The Pingdingshan Coalfield is located in the west-central region of Henan Province in northern China, with a length of about 40 km from east to west and a width of 20 km from north to south, covering an area of about 800 km^2^, and its geographic location is shown in Fig. 2. The coal-bearing strata in the Pingdingshan Coalfield are Late Paleozoic Permian coal-bearing rock systems.

The low, steep terrain is where the Pingdingshan Coalfield is situated. Additionally, the Guoshan Fault divides it into east and west regions. The primary water-filled aquifers in the Pingdingshan mine area are the Carboniferous Graywacke aquifer (L2 and L7) and the Cambrian Graywacke aquifer. Surface water, loose pore water of the Quaternary system, and water of the Permian Pingdingshan sandstone also have a direct and indirect impact on the mine’s extraction. Therefore, it is of great theoretical and practical significance to study the differences in the hydrochemical characteristics of various water filling sources, determine their typical ions, and establish corresponding identification models for timely judgment of mine water sources.

## Data analysis and processing

In the Pingdingshan coalfield, 124 sets of mine water source data were collected from February 2017 through December 2021. The source of the data is the Institute of Water Sciences, Henan polytechnic university. The concentrations of the main chemical discriminators Na^+^ + K^+^, Ca^2+^, Mg^2+^, SO_4_^2−^, Cl^−^ and HCO_3_^−^ were extracted using Shimadzu Ion Chromatography and ICP-MS with an error of 1% in the Key Laboratory of the School of Resource and Environment, Henan Polytechnic University, and the concentration of HCO_3_^−^ was determined by titration with dilute sulfuric acid methyl orange. A sample database was established based on this data, which includes 19 groups of Surface water (I), 16 groups of Quaternary pore water (II), 44 groups of Carboniferous limestone karst water (III), 22 groups of Permian sandstone water (IV) and 23 groups of Cambrian limestone karst water (V). Sample statistics for each aquifer are integrated in Table 1.

**Table 1:** Statistics of water source types in Pingdingshan.

Type of water source	Sample capacity	Label	One-hot encoding
Surface water	19	0	[1. 0. 0. 0. 0]
Quaternary pore water	16	1	[0. 1. 0. 0. 0]
Carboniferous limestone karst water	44	2	[0. 0. 1. 0. 0]
Permian sandstone water	22	3	[0. 0. 0. 1. 0]
Cambrian limestone karst water	23	4	[0. 0. 0. 0. 1]

Since the model cannot handle character-based data, the water type needs to be mapped to numeric data. Such a mapping would impose a magnitude relationship between water source types, Cambrian limestone lava water > Permian sandstone water > Carboniferous limestone lava water > Quaternary pore water. But no such relationship exists among water sources. To address this problem, a new virtual feature can be created using one-hot encoding, where a column of the virtual feature represents a value reflecting the size relationship between water sources. The implementation of the one-hot coding is based on Sklearn’s OneHotEncoder function. The type of sudden water is numerically labeled by LabelEncoder function to generate the coding dictionary {Surface water: 0, Quaternary pore water: 1, Carboniferous limestone water: 2, Permian sandstone water: 3, Cambrian limestone water: 4}. The code is expressed through four binary digits, belonging to the kind of water source that takes the value of 1, and vice versa is 0, such as surface water [1.0.0.0.0].

**Figure 3:**
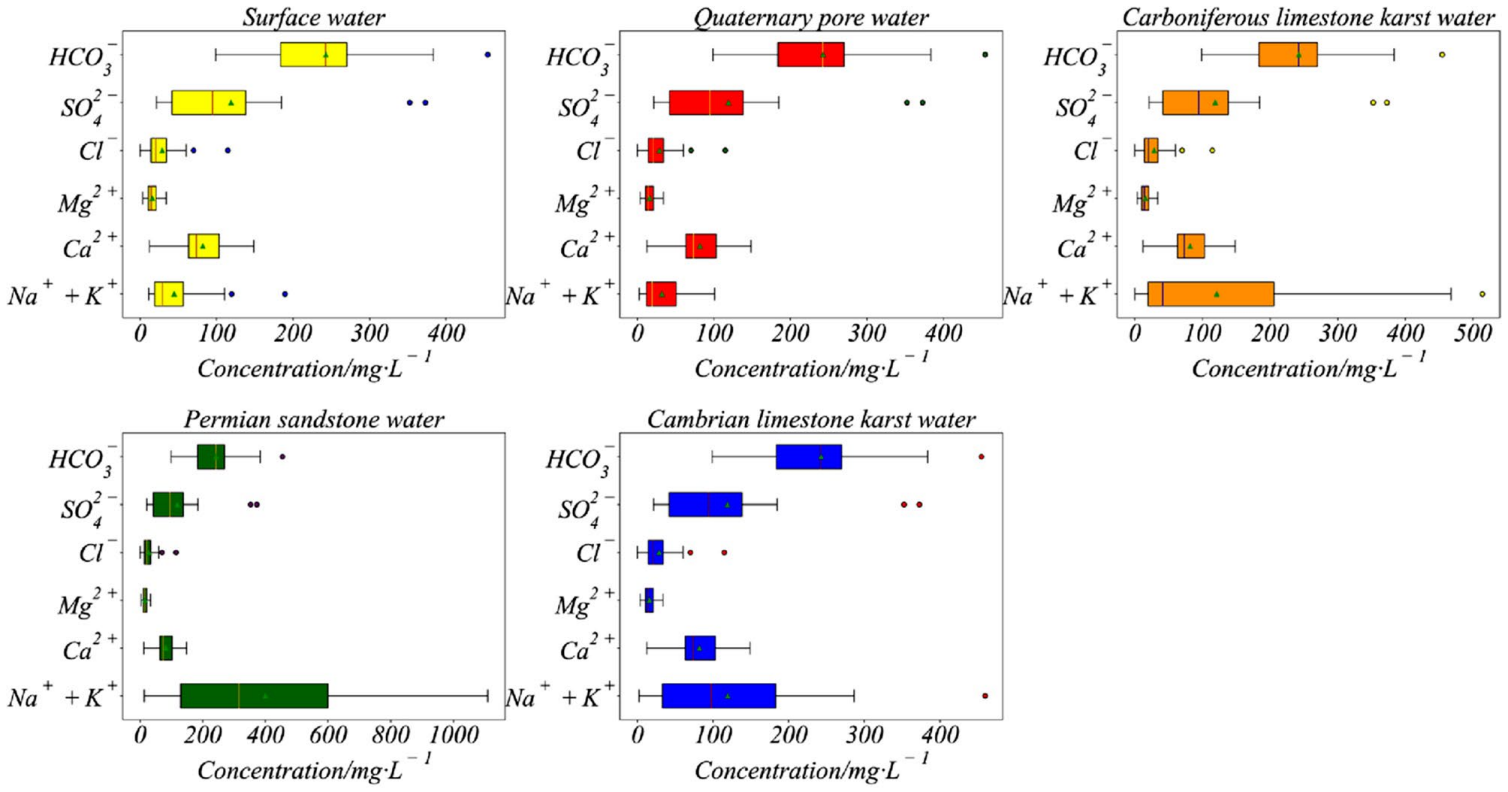
Box diagram of ion content in a water source sample.

The box plots in Fig. 3 clearly show the distribution of ion concentrations in different aquifers. In all aquifers, HCO^3−^ has the highest concentration distribution range compared to other ions, and Mg^2+^ has the lowest distribution range. The following shows the distribution of cation concentrations in Surface water, Quaternary pore water, and Carboniferous limestone karst water: Ca^2+^ > Na^+^ + K^+^ > Mg^2+^, the anion HCO_3_^−^ was the highest, and Cl^−^ was the lowest. But Na^+^ + K^+^ dominates, followed by Ca^2+^ and Mg^2+^ among the cations in Permian sandstone water and Cambrian limestone karst water. Permian sandstone water and Cambrian limestone karst water have similar anion concentrations, excessive HCO_3_^−^ concentrations, and similar SO_4_^2−^ and Cl^−^ distributions.

When the variability in the distribution of the variables is large, it tends to affect the model performance if left untreated. To eliminate the effect of the scale between input variables, the data need to be standardized to address the issue of comparability between indicators. The standardization of the deviation method, which can be computed using the following formula, is frequently used to handle the data:

9 $$ x^{\prime} = \frac{x - \min (x)}{{\max (x) - \min (x)}}, $$

where x is the current data, min(x) the minimum value of the current data and max(x) the maximum value of the current data and $\text{x}$′ is the normalized data.

In addition, box plots can reflect outlier samples. Idiosyncratic points in the sample, or points with high residuals, that vary considerably from the range of the data distribution are called outliers. These outliers are true data, and there might be a faulty piece of experimental apparatus to blame for the stark disparity in findings, redox reactions during chemical ion extraction, and microbial action. When a sample point in a box plot goes below the lower quartile and exceeds the higher quartile, it is considered an outlier. Twenty sets of samples that were above the threshold were deleted by Fig. 3, which is represented as a diamond. Table 2 displays the outlier samples.

**Table 2:** Outlier sample statistics.

Number	Ion(mg·L^−1^)						Category
	K^+^ + Na^+^	Ca^2+^	Mg^2+^	Cl^−^	SO_4_^2−^	HCO_3_^−^	
3	110.86	119.64	27.22	114.86	352.54	149.5	I
48	189.52	12.42	9.11	48.21	125.36	344.15	I
49	26.22	141.57	34.09	0	372.82	181.23	I
83	6.44	199.4	61.97	124.78	107.59	583.96	II
121	47.61	380.56	58.44	117.34	587.41	630.34	II
131	11.15	122.24	27.76	48.11	46.18	391.33	II
60	513.99	14.56	1.1	101.82	57.94	1074.77	III
61	369.84	49.7	24.18	61.68	524.97	480.84	III
95	93.11	5.61	72.84	52.47	130.14	360.02	III
138	34.11	88.99	13.27	26.16	64.41	289.41	III
140	29.58	106.54	19.71	27.83	101.27	284.96	III
149	400.22	21.77	7.99	91.68	53.01	817.4	III
150	374.22	22.48	13.74	92.47	102.95	809.11	III
8	809.6	4.8	15.07	151.02	143.12	1796.42	IV
13	158.24	167.13	173.4	30.49	1335.23	52.48	IV
50	776.71	383.77	125.39	114.86	249.36	497.31	IV
53	807.37	14.48	21.2	47.68	825.67	1021.91	IV
39	59.87	417.33	21.71	88.63	937.35	180.84	V
42	7.05	172.34	81.47	208.74	131.17	460.85	V
93	459.6	6.09	8.34	72.77	121.3	928.3	V

## TPE-LightGBM water source discrimination model

**Figure 4:**
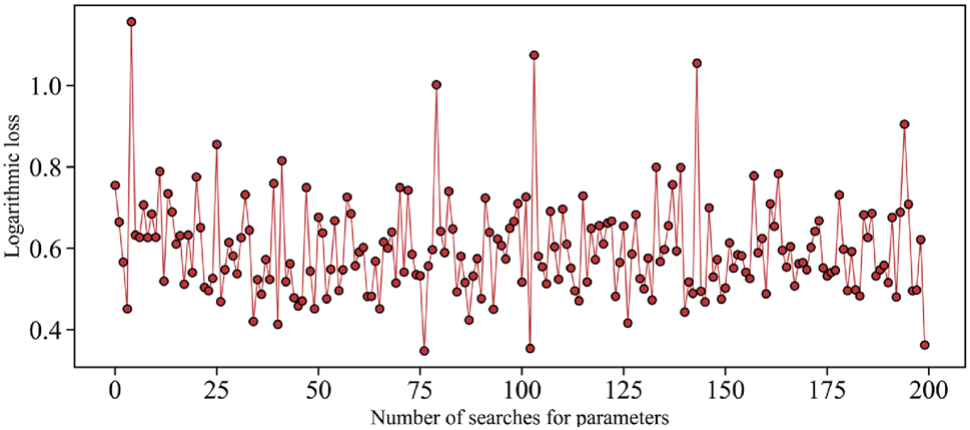
Curve of logarithmic loss value changing with parameter seeking times.

This experiment is mainly implemented programmatically in the Spyder platform, with TPE based on the Hyperopt function library and LightGBM built from the Sklearn machine learning library. The model construction process is as follows:

- *Step 1* The processed samples are divided into 80% learning samples and 20% test samples. The learning samples are for the model to learn, and the test samples evaluate the model’s performance. Initialize the LightGBM model.
- *Step 2* Set the number of searching parameters of TPE to 200 and the searching range. Find the optimal values for the six parameters of Learning_rate, Max_depth, N_estimators, Num_leaves, Feature_fraction, and Max_bin of LightGBM. The parameter space is displayed in Table 3.
- *Step 3* After 200 iterations of parameter searching, the TPE obtains the optimal solution, and the outcomes are displayed in Table 3. TPE selects the optimal parameters based on the log-likelihood loss values. As the number of times increases, it is evident that the loss value looks to be minimum at around 75 times, and the minimal value from Fig. 4 is approximately 0.342. At this point, the inverse solution function achieves an optimal solution. The red dots in the figure represent the logarithmic losses corresponding to a certain number of parameter searches.
- *Step 4* The optimal combination of parameters obtained is back substituted for the LightGBM model to verify the effectiveness of the TPE algorithm and the merits of the TPE-LightGBM performance.

**Table 3:** Parameter seeking range of TPE algorithm.

Parameter	Importance	Parameter scale	TPE result
Learning_rate	High	[0.01, 0.5]	0.46
Max_depth	High	[0, 6]	4
N_estimators	Moderate	[1, 300]	113
Num_leaves	High	[1, 300]	172
Feature_fraction	Low	[0.6, 1]	0.9
Max_bin	High	[1, 200]	177

**Figure 5:**
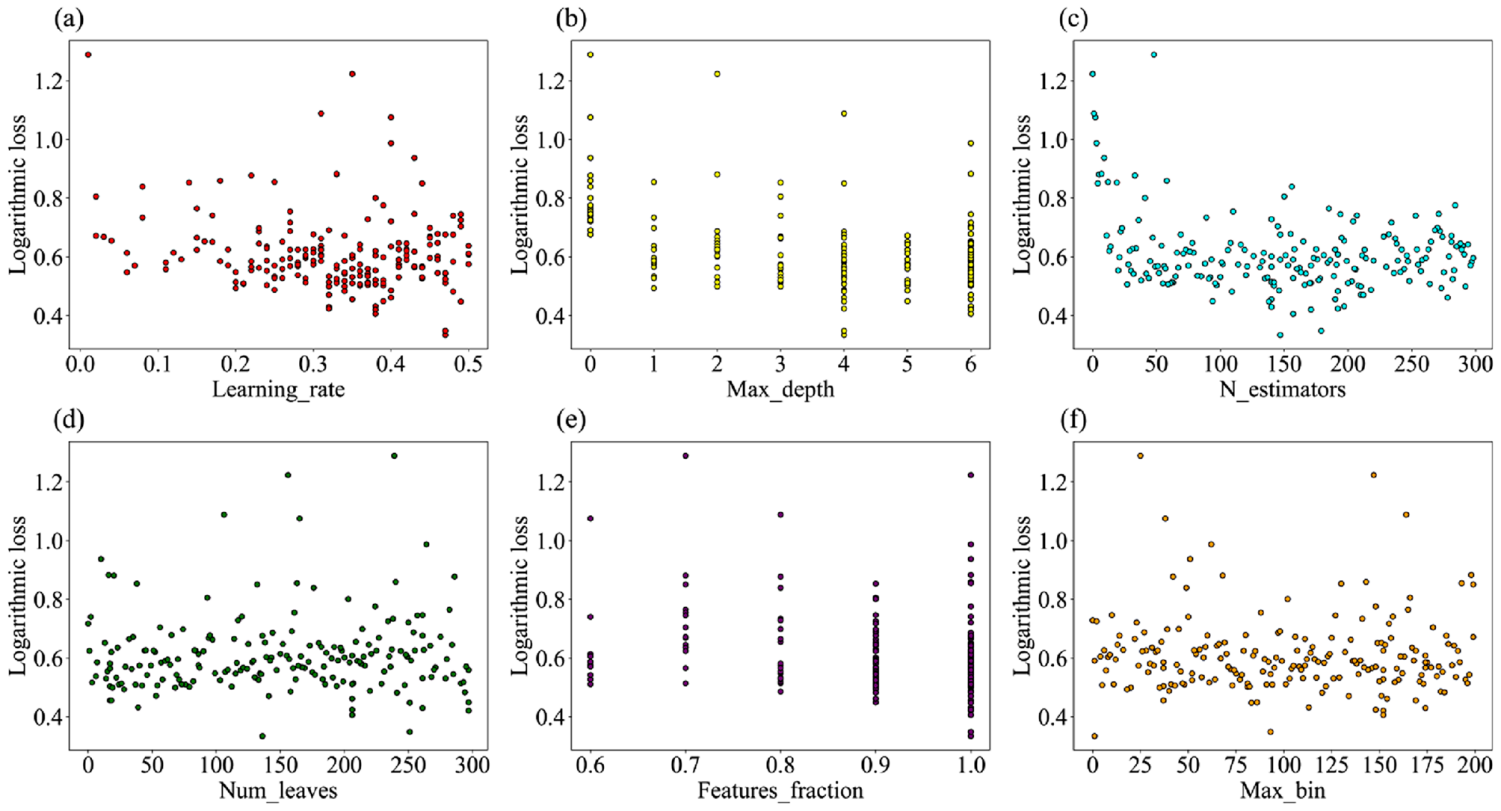
Parameter selection process of TPE optimization.

Currently, numerous algorithms for machine learning are a black box. However, hyperopt can get the results of the search process by passing in Trials to get a better understanding of how the parameters relate to the model. The parameter values are continually fluctuating, as Fig. 5a illustrates, and the TPE is gradually gaining knowledge about the effects of the parameters. Learning_rate is equivalent to a loss value that is concentrated in the area that is close to 0. It is evident from the graphic that it accepts parameter values between 0.4 and 0.5. When max_depth = 4, as seen in Fig. 5b, the loss value reaches its lowest value; in Fig. 5c, N_estimators determine the lowest value of loss to be approximately 150. The method ultimately yields a number of 113, though, which would indicate that some parameter combinations produce superior results. Similar approaches might be used to find the optimal values for Num_leaves, feature_fraction, and Max_bin in Fig. 5d–f.

### Model performance evaluation

**Figure 6:**
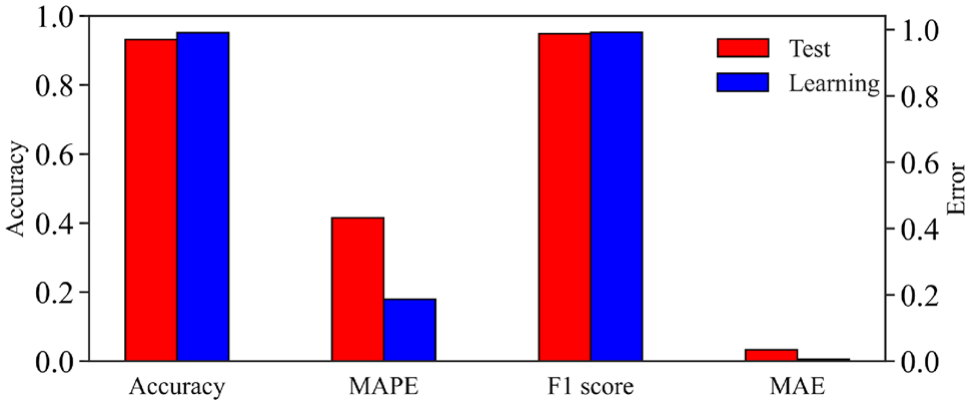
Model performance analysis diagram.

Machine learning evaluates a classification model by calculating some metrics of the model, such as accuracy, root-mean-square error (RMSE), F1 score (Micro-F1), and mean absolute error (MAE). The hyperparameters are substituted into the model to determine whether the model is superior or inferior based on its performance in the test sample versus the learning sample. As shown in Fig. 6, the model has an accuracy of 0.931 for the test sample and an RMSE of only 0.187 for the training sample. The trained water source identification models were well-fitted and did not show overfitting and underfitting. In addition, the F1 score of the model is close to 1, and there is almost no generalization error, proving that the model performs well.

To further validate the model’s dependability, TPE-LightGBM is designed to be compared with the commonly used machine learning classification methods multilayer perceptron machine (MLP) and support vector machine (SVM). In addition, crucial parameters of MLP and SVM are optimized by random search (RS) and genetic algorithm (GA). The parameter search spaces of MLP and SVM algorithms are shown in Tables 4 and 5 respectively. RS optimizes the parameters such as the number of hidden layers, the number of neurons per layer, the learning rate, and the step size of the MLP. GA tunes SVM parameters such as Penalty factor (C), Gamma and Kernel. The search times of the three automatic parameter-finding methods are compared, and the results are shown in Table 6. Among them, TPE guarantees the shortest time despite the high number of times, followed by GA. RS spends the most time.

**Table 4:** Parameter seeking range of MLP algorithm.

Parameter	Importance	Parameter scale	Result
Hidden_layer_sizes	High	[1, 5]	4
Number of neurons	High	[20, 100]	80
Learning_rate_init	High	[0.001, 1]	0.001
Batch_size	Moderate	(2^2^, 2^3^, 2^4^, 2^5^, 2^6^)	4

**Table 5:** Parameter seeking range of SVM algorithm.

Parameter	Importance	Parameter scale	Result
C	High	[0, 1]	1
Gamma	High	[‘Auto’, ‘Scale’]	Auto
Kernel	Moderate	[‘Rbf’, ‘Poly’, ‘Sigmoid’]	Rbf

**Table 6:** Comparison of efficiency of three parameter seeking methods.

Method	Parameter seeking frequency	Time (s)
TPE	200	47.63
RS	120	783.41
GA	50	114.48

**Figure 7:**
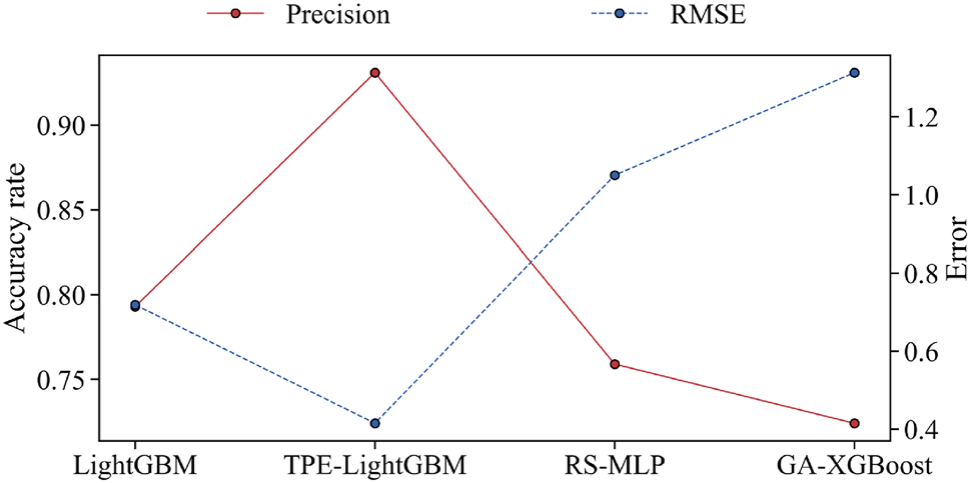
Performance comparison of the three models.

To maintain objectivity, the TPE-LightGBM, RS-MLP, and GA-SVM models are successively trained using the same batch of learning examples. The three models’ accuracy and generalization errors in the test samples serve as the assessment metrics. As shown in Fig. 7, the accuracies of LightGBM, TPE-LightGBM, RS-MLP, and GA-SVM are 0.793, 0.931, 0.759, and 0.724 in that order. RS-MLP has the lowest accuracy and higher generalization error, which suggests that the RS-MLP model is a weak fit for the learning samples and the tree model is a better fit for small and medium-sized data. The LightGBM model without TPE optimization performs about the same as the GA-SVM model. However, the TPE-optimized LightGBM model’s accuracy increased by 0.139, which proves the effectiveness of the TPE method.

### Analysis of the contribution of variables

**Figure 8:**
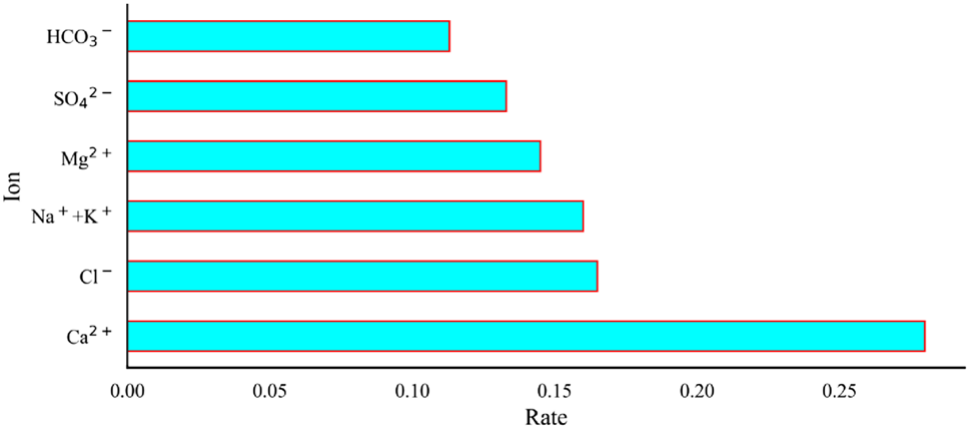
The degree of influence of ionic variables on decision making results.

To reflect the ability of each ion of the aquifer to discriminate the source of sudden water^7^, the variable contribution was quantified by recording the total number of splits of the root node and the average information gain. The variables were ranked in terms of importance based on the magnitude of the calculated Gini coefficients. The formula for the Gini coefficient will not be repeated here. The Gini coefficient for each chemical ion can be calculated from Equation: ${\text{VIM}}_{{{\text{Ca}}^{2 + } }}^{{\left( {{\text{Gini}}} \right)}} = 0.28,{\text{ VIM}}_{{{\text{Cl}}^{ - } }}^{{\left( {{\text{Gini}}} \right)}} = 0.165,{\text{ VIM}}_{{{\text{Na}}^{ + } + {\text{K}}^{ + } }}^{{\left( {{\text{Gini}}} \right)}} = 0.16,{\text{ VIM}}_{{{\text{Mg}}^{2 + } }}^{{\left( {{\text{Gini}}} \right)}} = 0.145,{\text{ VIM}}_{{{\text{SO}}_{4}^{2 - } }}^{{\left( {{\text{Gini}}} \right)}} = 0.133,{\text{ VIM}}_{{{\text{HCO}}_{3}^{ - } }}^{{\left( {{\text{Gini}}} \right)}} = 0.113.$ The magnitude of the Gini coefficient determines how much the variable affects the model results. As can be seen in Fig. 8, Ca^2+^ has the greatest influence on the results and HCO^3−^ has the least significance on the results. Water chemistry ions are ranked by significance: Ca^2+^ > Cl^−^ > Na^+^ + K^+^ > Mg^2+^ > SO_4_^2−^ > HCO_3_^−^.

### Model validation

**Figure 9:**
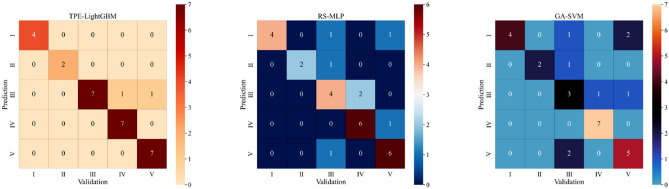
Confusion matrix diagram of TPE-LightGBM, RS-MLP and GA-SVM.

In this study, 29 groups of unknown water sources in Pingdingshan were selected as validation samples. The validation samples consisted of four groups of surface water, two groups from the Quaternary aquifer, seven groups from the Carboniferous aquifer, eight groups from the Permian aquifer, and eight groups from the Cambrian aquifer. The confusion matrices of TPE-LightGBM, RS-MLP, and GA-SVM for validation sample identification are shown in Fig. 9. The numbers in the diagonal position represent the number of samples correctly identified by the corresponding water source type, and the numbers in the non-diagonal position represent the number of samples incorrectly identified by the corresponding water source type. TPE-LightGBM misidentified only one Permian aquifer and one Cambrian aquifer as Carboniferous aquifer. Misidentification may be due to the proximity of the sampling location of the Carboniferous aquifer to the other two types of water sources. They may be conductive through fissures or faults. Therefore, they have similar ion concentrations. However, the other two models show numerous errors in the identification of validation samples, and it can be argued that TPE-LightGBM outperforms these two models. The TPE-LightGBM has an even greater advantage in source identification issues.

## Conclusion and outlook

The work proposes an intelligent water source classification model based on TPE-LightGBM, which provides a new approach to mine water source identification. The main results are as follows:

1. Twenty sets of anomalous samples were identified and rejected through box plots reflecting trends in the distribution of water chemistry data. Potential problems with the data are addressed before training, thus reducing the probability of overfitting of the model.
2. The TPE algorithm optimizes the main parameters of LightGBM. Upon comparing the pre- and post-optimization data, TPE was shown to have enhanced LightGBM’s performance by 13.9%, which shows the effectiveness and applicability of the TPE algorithm to the LightGBM model. Then, Comparing TPE-LightGBM with commonly used machine learning algorithms such as RS-MLP and GA-SVM, TPE-LightGBM has a higher accuracy of 93.1%.
3. Based on the calculation of Gini coefficients between the variables, it can be obtained that Ca^2+^ has the highest Gini coefficient, indicating that Ca^2+^ has a large contribution to the model prediction. Therefore, changes in Ca^2+^ concentration should be noted in the extraction of chemical data.

The model has not been applied to the mining area with the same hydrogeological type as Pingdingshan mining area, and there are some limitations. The sample concentration exceeds the variable concentration range of the training sample, which has a certain impact on the recognition accuracy. Future research should collect more data, build a sample database, and improve the generalization performance of the model. It is hoped that future studies will take these issues into account.

## Data Availability

The data that support the findings of this study is available from the Institute of Water Science but restrictions apply to the availability of this data, which was used under license for the current study, and so is not publicly available. Data is however available from the author Zhenwei Yang upon reasonable request and with permission of the Institute of Water Science.
